# Effects of Unfiltered Coffee and Bioactive Coffee Compounds on the Development of Metabolic Syndrome Components in a High-Fat-/High-Fructose-Fed Rat Model

**DOI:** 10.3390/nu10101547

**Published:** 2018-10-19

**Authors:** Pedram Shokouh, Per Bendix Jeppesen, Kjeld Hermansen, Christoffer Laustsen, Hans Stødkilde-Jørgensen, Stephen Jacques Hamilton-Dutoit, Mette Søndergaard Schmedes, Haiyun Qi, Thomas Stokholm Nørlinger, Søren Gregersen

**Affiliations:** 1 Department of Endocrinology and Internal Medicine, Aarhus University Hospital, Palle Juul Jensens Boulevard 165, 8200 Aarhus N, Denmark; per.bendix.jeppesen@clin.au.dk (P.B.J.); kjeld.hermansen@aarhus.rm.dk (K.H.); soeren.gregersen@aarhus.rm.dk (S.G.); 2 The Danish Diabetes Academy, Odense University Hospital, Kløvervænget 10, 5000 Odense, Denmark; 3 MR Research Centre, Aarhus University Hospital Skejby, Palle Juul Jensens Boulevard 99, 8200 Aarhus N, Denmark; cl@clin.au.dk (C.L.); hsj@clin.au.dk (H.S.-J.); qi@clin.au.dk (H.Q.); thomasnorlinger@gmail.com (T.S.N.); 4 Institute of Pathology, Aarhus University Hospital Skejby, Palle Juul Jensens Boulevard 99, 8200 Aarhus N, Denmark; stephami@rm.dk; 5 Department of Food Science, Aarhus University, 5792 Årslev, Denmark; mette.schmedes@food.au.dk

**Keywords:** coffee, chlorogenic acid, caffeic acid, trigonelline, insulin resistance, metabolic syndrome x, non-alcoholic fatty liver disease, carbon-13 magnetic resonance spectroscopy, phytotherapy

## Abstract

The literature is inconsistent as to how coffee affects metabolic syndrome (MetS), and which bioactive compounds are responsible for its metabolic effects. This study aimed to evaluate the effects of unfiltered coffee on diet-induced MetS and investigate whether or not phenolic acids and trigonelline are the main bioactive compounds in coffee. Twenty-four male Sprague‒Dawley rats were fed a high-fat (35% W/W) diet plus 20% W/W fructose in drinking water for 14 weeks, and were randomized into three groups: control, coffee, or nutraceuticals (5-*O*-caffeoylquinic acid, caffeic acid, and trigonelline). Coffee or nutraceuticals were provided in drinking water at a dosage equal to 4 cups/day in a human. Compared to the controls, total food intake (*p* = 0.023) and mean body weight at endpoint (*p =* 0.016) and estimated average plasma glucose (*p* = 0.041) were lower only in the coffee group. Surrogate measures of insulin resistance including the overall fasting insulin (*p* = 0.010), endpoint HOMA-IR (*p* = 0.022), and oral glucose tolerance (*p* = 0.029) were improved in the coffee group. Circulating triglyceride levels were lower (*p* = 0.010), and histopathological and quantitative (*p* = 0.010) measurements indicated lower grades of liver steatosis compared to controls after long-term coffee consumption. In conclusion, a combination of phenolic acids and trigonelline was not as effective as coffee per se in improving the components of the MetS. This points to the role of other coffee chemicals and a potential synergism between compounds.

## 1. Introduction

Metabolic syndrome (MetS) encompasses a constellation of cardiometabolic risk factors, i.e., visceral adiposity, glucose intolerance, elevated blood pressure, and dyslipidemia, defined to help identify people at increased risk of cardiovascular diseases and type-2 diabetes mellitus (T2D) [1]. Long-term follow-up studies have indicated that MetS can also predict the future risk of cardiovascular mortality [2]. Visceral adiposity and the concomitant insulin resistance (IR) are the driving forces behind the metabolic derangements of the MetS [3]. Since this syndrome affects 25% of the world’s population [4], effective preventive measures will confer immense public health benefits.

The effect of coffee on health has historically been a controversial topic. However, evidence is now convincing that coffee consumption diminishes the risk of all-cause mortality as well as several types of cancer, cardiovascular disease, chronic liver disease, Parkinson’s disease, and T2D, while the risk of urinary tract and lung cancer can slightly be increased [5]. The potential role of coffee in the prevention of T2D was originally identified through observational research [6]. To understand how coffee affects the risk of T2D, it is necessary to study its effects on T2D risk factors clustered in the definition of MetS. Epidemiological data show a non-linear reverse relationship between coffee intake and risk of MetS [7] and its hepatic component, non-alcoholic fatty liver disease (NAFLD) [8]. NAFLD is a manifestation of ectopic fat deposition, which is strongly associated with all features of the MetS [9]. As yet, long-term interventional research on the effects of coffee on different features of the MetS is lacking.

While coffee is a blend of more than 1000 volatile and non-volatile compounds, only a minority reach bioactive concentrations with moderate daily consumption, comprising alkaloids (including caffeine and trigonelline), phenolic acids (including chlorogenic acids, CGA), diterpenes (including cafestol and kahweol), and Maillard reaction and degradation products formed during the roasting process (including melanoidins and quinides) [10]. However, the debate still continues as to which compound or combination of compounds explains the preventive potential of coffee on T2D risk factors.

The present study aimed to assess the long-term effects of a realistic dosage of unfiltered coffee on the development of the key features of MetS and to compare coffee with a combination of its main bioactive compounds. The second goal was to determine whether or not phenolic acids and trigonelline are the main compounds behind the metabolic efficacy of coffee in MetS subjects. A combination rather than single compounds was used in the study based on the hypothesis that synergy plays an essential role in the biological effects of coffee.

## 2. Materials and Methods

### 2.1. Animal Model

Six-week-old male Sprague‒Dawley^®^ rats (*n* = 24) were purchased from Taconic A/S (Ejby, Denmark). For the first week, rats were caged individually and allowed to acclimatize with free access to regular rat chow (Altromin 1324, Altromin GmbH, Lage, Germany). After acclimatization, ad libitum high-fat diet (D12492, Research Diets, Inc., New Brunswick, NJ, USA) was introduced and simultaneously, the intervention started. The regular chow contained 4.1% W/W fat (11% of total calories), 19.2% W/W protein (24% of total calories), 59.5% W/W carbohydrate (65% of total calories), and the high-fat diet contained 34.9% W/W fat (60% of total calories), 26.2% W/W protein (20% of total calories), and 26.3% W/W carbohydrate (20% of total calories). The nutrient composition of the high-fat diet used in this study is presented in the Table A1. D-(−)-Fructose (Sigma-Aldrich, St. Louis, MO, USA) was also given (~9 g/day/rat) in drinking water. Since the majority of rats were consuming their entire fixed dosage of fructose solution, the daily fluid consumption was not monitored in this study.

Rats were kept under reversed 12-h light‒dark cycles at temperatures between 21 and 24 °C and 55–70% relative humidity. Animals were housed and handled by qualified personnel in keeping with EU and Danish provisions under regular random inspections. The study protocol was approved by the Animal Experimentation Council of the Danish Animal Experiments Inspectorate and given the registration number 2015-15-0201-00592.

### 2.2. Experimental Design

The experiment consisted of one control and two intervention groups receiving either unfiltered brewed coffee or selected coffee compounds (referred to as the nutraceuticals group) in drinking water for 14 weeks. Eight rats were randomly allocated into each group.

At baseline, body weight and daily food consumption were measured, and a fasting tail blood sample was collected. Weighing of all rats was repeated every week, food consumption monitoring biweekly, and tail blood sampling monthly. On week 12, nuclear magnetic resonance (MR) imaging was performed (described later in this section). After one week of recovery, rats underwent an oral glucose tolerance test (OGTT) by administering 2 g/kg of D-glucose via gastric gavage after 8 h of fasting and measuring the tail blood glucose by a FreeStyle Precision glucometer (Abbott GmbH & Co. KG, Wiesbaden, Germany) before and 30, 60, 120, and 180 min after the gavage [11]. At the end of the study, rats were deeply anesthetized by intraperitoneal injection of 50 mg/kg of pentobarbital and blood samples were obtained from the retro-orbital plexus and urine samples from the bladder. Liver and pancreas were harvested and fixed by immersion in 4% formaldehyde and stored at 4 °C until paraffin embedding (see the histopathological examination section). During the six to eight hours of fasting time before blood sampling, OGTT, and surgery, a drinking solution without fructose was given to the animals. At the end of the experiments, rats were terminated by exsanguination.

### 2.3. Intervention

Coffee was brewed by a French press coffee maker with a boiling water to ground coffee proportion of 180 mL to 8 g for making 150 mL of brewed coffee. Fructose was then added to the beverage and it was diluted with water 2.3 times to make the final drinking solution. On average, each rat received coffee made from ~1.18 g of ground medium-roast Ethiopian Arabica coffee (Hans & Grethe Coffee-Tea, Aarhus, Denmark) per day. For the nutraceuticals group, the sum of chemicals (24 mg of CGA, 12 mg of caffeic acid, and 7 mg of trigonelline per rat per day) was dissolved in 5 mL of 70% ethanol and added to the fructose solution. The same amount of ethanol was also given to the other groups. Of the at least 69 known isomers of CGAs present in coffee [12], 3-, 4-, and 5-*O*-caffeoylquinic acids (CQAs) are the most abundant. 5-CQA was used at a dosage (24 mg/day/rat) equal to the total CQAs content of four cups of brewed medium-roast Ethiopian Arabica coffee (around 155 mg per cup [13]) in an adult. Twelve milligrams per day of caffeic acid were also added to the combination to compensate for the absence of the other groups of phenolic acids. The third constituent of this coffee-based supplement was trigonelline, an alkaloid present in roasted coffee in amounts averaging around 6.2 mg/g of ground beans [14]. The trigonelline present in four cups of coffee per day in a human is equivalent to 7 mg/day in an average rat. Trigonelline is known to exert in vivo glucose- and lipid-lowering effects [15]. The chemical composition of the coffee brew used in this study had not been analyzed. Instead, we have calculated the dosage of nutraceuticals based on published analyses of the same type of coffee as stated above. Chlorogenic acid was purchased from MP Biomedicals (Santa Ana, CA, USA) and trigonelline hydrochloride and caffeic acid from Sigma Aldrich.

There were two main reasons for choosing this combination of nutraceuticals: (1) Besides caffeine and diterpenoids, trigonelline and CGAs are assumed to be the main compounds present in bioactive concentrations in coffee brew, while the concentration of other classes of nutraceuticals such as isoflavones [16] and flavan-3-ols [17] is trivial. Since epidemiological [18,19] and experimental [20,21] evidence has indicated that both caffeinated and decaffeinated coffee are effective in T2D and MetS prevention, and uncertainty still exists around the long-term effects of caffeine on insulin sensitivity, caffeine was not incorporated into the tested combination. Moreover, diterpenoids were excluded due to the fact that, at the time of study design, there was no clue as to whether these compounds may improve the efficacy of the combination. (2) A number of presumably bioactive roasting process products, e.g., CGA lactones, melanoidins, and *N*-methylpyridinium, are not commercially available.

Intervention dosage in both groups was equivalent to four cups of coffee in an average adult. This dosage is realistic since it falls into the range of moderate coffee consumption, typically defined as 3‒5 cups per day, based on the European Food Safety Authority’s review of caffeine safety [22]. The dose conversion was performed according to the body surface area (BSA) normalization method [23] with the following premises: rat BSA = 0.06 m^2^ and human BSA = 1.69 m^2^. BSA was estimated using the equation by Haycock et al. [24]: BSA (m^2^) = 0.024265 × height^0.3964^ (cm) × weight^0.5378^ (kg). The drinking solution was dispensed in fixed amounts every other day to all groups in order to harmonize their fructose intake and intervention dosages.

### 2.4. Immunoassay Analyses

Glucose, lipoproteins, and alanine aminotransferase (ALT) in plasma and glycated hemoglobin (HbA1C) in whole blood samples (collected in EDTA tubes) were quantified by Cobas c111 analyzer (Roche Diagnostics, Mannheim, Germany). A sensitive rat RIA kit (EMD Millipore, Billerica, MA, USA) was used to measure insulin. NEFA-HR2 assay (Wako Chemicals GmbH, Neuss, Germany) was utilized to determine non-esterified fatty acids (NEFAs) concentration. Plasma total antioxidant capacity was quantified by a commercial assay kit (MAK 187, Sigma Aldrich, Steinheim, Germany) according to the manufacturer’s instructions. Liver triglycerides content was estimated by cutting around 50 mg of frozen liver tissue per sample on dry ice and obtaining its saponified extract using a published protocol [25]. Triglyceride concentration was then quantified by a Cobas c111 analyzer.

### 2.5. Histological Examinations

The right anterior lobe of the liver was dissected and fixed in 10% neutral buffered formalin. Fixed tissues were then embedded in paraffin and cut into 4-mm slices and stained with hematoxylin and eosin, and Masson-trichrome. The blinded examination was performed on a random section from each animal by scanning at low power and then scrutinizing it in detail in five medium-power fields. The sections were photographed under 100× magnification. Steatosis was divided into three types: large-droplet macrovesicular, small-droplet macrovesicular, and microvesicular. Each type was further graded on a scale of 0‒3 based on the percentage of hepatocytes containing fat vacuoles: grade 0 < 5%, grade 1 = 5–33%, grade 2 = 34–66%, and grade 3 > 67%. The sections were additionally evaluated for hepatocyte ballooning, inflammation, and fibrosis.

### 2.6. Hyperpolarized-[1-^13^C]-Pyruvate MR Spectroscopy

Rats with a weight range of 400 to 660 g were scanned in a 3T GE clinical system (GE Healthcare, Milwaukee, WI, USA) equipped with a dual tuned ^13^C/^1^H volume rat coil (GE healthcare, Brøndby, DK). Hyperpolarized [1-^13^C]-pyruvate was prepared and polarized in a SpinLab system (GE Healthcare) in accordance with the standard protocol [26]. In brief, rats were anesthetized with sevoflurane (3% sevoflurane in 2 L/min air) and a tail vein catheter (0.4 mm) was inserted for injection of [1-^13^C]-pyruvate, upon full polarization (>35%) 1.5 mL (37 °C, pH 7.4) isotonic [1-^13^C]-pyruvate solution was injected over 15 s. Anatomical ^1^H imaging used for positioning the ^13^C imaging plane a T2-weighted fast spin echo sequence was used in the axial and coronal orientation covering the liver. Following the anatomical scout, an axial oblique slice-selective (10 mm, 10°) ^13^C-dynamic time series with a repetition time of 1 s (total 120 s, one image/s). The sequence was initiated before the injection of [1-^13^C]-pyruvate. The individual peak areas were fitted using a general linear model fit on the time domain data, followed by a model-free ratiometric analysis of the area under the curve (AUC) of product and substrates.

### 2.7. ^1^H MR Spectroscopy

Urine samples were thawed at room temperature, vortexed for 30 s and centrifuged (10,000 rpm for 5 min). A total of 100–400 μL urine was transferred from the supernatant into a 5-mm MR tube (VWR International, West Chester, PA, USA) and mixed with 100 μL 0.75 M phosphate buffer solution containing 0.5% TSP prepared in D_2_O. All MR spectra were recorded at 298 K on a Bruker Avance 600 spectrometer operating at a ^1^H MR frequency of 600.13 MHz and equipped with a 5 mm TXI probe (Bruker BioSpin, Rheinstetten, Germany). The Bruker ‘zgpr’ pulse program was applied as a pre-saturation pulse sequence for water suppression. A total of 64 scans were collected into 32 K data points with a relaxation delay of 2 s. MR spectra were phased and baseline-corrected using TopSpin 3.0 software (Bruker BioSpin, Rheinstetten, Germany). The MR peaks were assigned based on the Human Metabolome Database [27] and Chenomx MR Suite (Chenomx Inc., Edmonton, AB, Canada). Selected peaks were integrated relative to the total peak area using Chenomx MR Suite.

### 2.8. Statistical Methods

Data are presented as mean and standard deviation (SD) plus 95% confidence interval (CI), where appropriate. The normality of the data was checked using quantile-quantile (q-q) plots and a Shapiro‒Wilk normality test. Shapiro‒Wilk test has provided a secondary basis for evaluating the normality of data where q-q plots were not unequivocal. Study groups were compared by one-way analysis of variance (ANOVA) followed by group-wise post hoc comparisons. A protected Fisher’s least significant difference (LSD) test was used as a valid method for three-group comparisons [28]. Where Levene’s test rejected the equality of variances, an F-test from the Welch’s test was used instead of one-way ANOVA, and Games‒Howell post hoc test was employed as an alternative to the LSD test. Trigonelline in the urine was compared between two intervention groups using an independent samples Student’s *t*-test. All the analyses were performed at a two-sided significance level of 0.05 using IBM SPSS Statistics for Windows (Version 22.0, IBM Corp, Armonk, NY, USA). AUC calculations were made using GraphPad Prism for Windows (version 5.01, GraphPad Software, La Jolla, CA, USA). The rate of changes of selected variables was calculated using the slope function of Microsoft Excel 2013 (Microsoft Corp., Redmond, WA, USA).

## 3. Results

### 3.1. Food Intake and Body Weight

**Figure 1:**
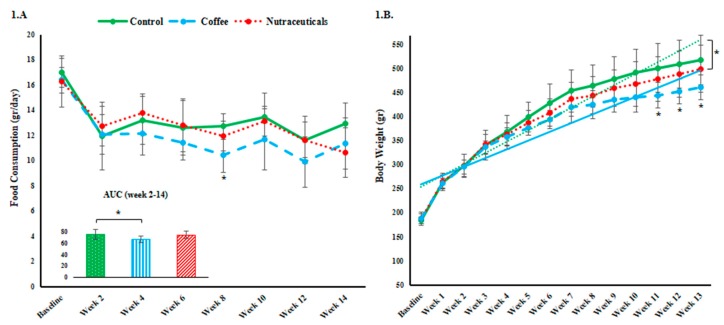
(**A**) The mean daily food consumption measured biweekly and the total food consumed between weeks 2 and 14 (as the area under the curve). (**B**) The trend of changes in body weight of each group over time is displayed. Straight lines represent the slope of changes in coffee vs. the control group; Error bars represent standard deviation of the mean. Asterisks denote significantly different values from the control group (* *p* ˂ 0.05).

Figure 1A shows an initially comparable but significant decline in food consumption (*p* < 0.01 in all groups) during the first two weeks due to an adapting response to the shift from a normal to high-fat/high-fructose (HFHF) diet. The overall food intake during the intervention period (AUC) was lower in the coffee group compared to the control group (post-hoc *p* = 0.023). Total food intake after week two (calculated by the area under the curve) was significantly reduced by coffee consumption as compared with the controls (post-hoc *p* = 0.023). From week five, the body weight of the rats in the coffee group started to split from the control rats with a significantly lower slope of increase (post-hoc *p* = 0.008). Slower weight gain led to significantly lower body weights in the coffee compared to the control group after week 10 (Figure 1B). The pure nutraceuticals mixture failed to replicate the brewed coffee’s effects.

### 3.2. Plasma Glucose and Insulin Resistance

Although slightly reduced fasting plasma glucose (FPG) in intervention groups at weeks 10 and 14 did not prove to be significant, long-term glycemic control measured by HbA1C was improved by coffee compared to both control (mean difference: −1.6 mmol/mol, 95% CI: −0.7 to −3.2 mmol/mol) and nutraceuticals groups (Table 1). Rats in the coffee group maintained lower fasting insulin levels compared to the other groups all along the intervention period and had the lowest AUC of fasting insulin (Table 1).

**Figure 2:**
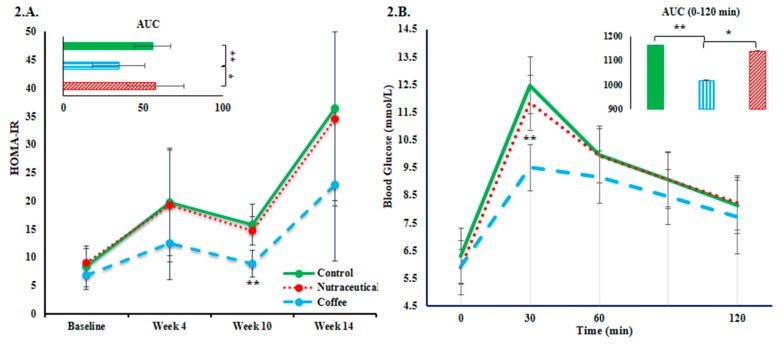
Surrogate measures of insulin resistance: (**A**) changes in HOMA-IR in each study group and its area under the curve (AUC); (**B**) repeated plasma glucose measurements during an oral glucose tolerance test and the AUC of changes in the first two hours. Error bars are standard deviation of the mean. Asterisks denote significantly different values (* *p* ˂ 0.05, ** *p* ˂ 0.01).

The overall IR estimated by the AUC of monthly HOMA-IR values was mitigated by coffee compared to both control and nutraceuticals groups (F-test *p* = 0.014 and *p* < 0.05 in post-hoc comparisons). Per-timepoint comparison showed lower HOMA-IR values in the coffee group at week 10 (F-test *p* < 0.001). (Figure 2A). The effect of coffee and nutraceuticals on glucose tolerance was compared after 13 weeks by a standard OGTT. According to the results displayed in Figure 2B, compared to the control group, plasma glucose peak at 30 min was on average 3.0 mmol/L (95% CI: 1.6–4.3 mmol/L) lower (*p* < 0.001), and area under the OGTT curve during the first two hours was significantly lower in the coffee group.

### 3.3. Plasma Lipid Profile

Mean plasma levels of different types of lipoproteins at endpoint are summarized in Table 2. Fasting NEFAs and major lipoprotein values were comparable between groups; however, triglycerides levels were reduced only by adding coffee to the HFHF diet.

### 3.4. Hepatic Steatosis

**Figure 3:**
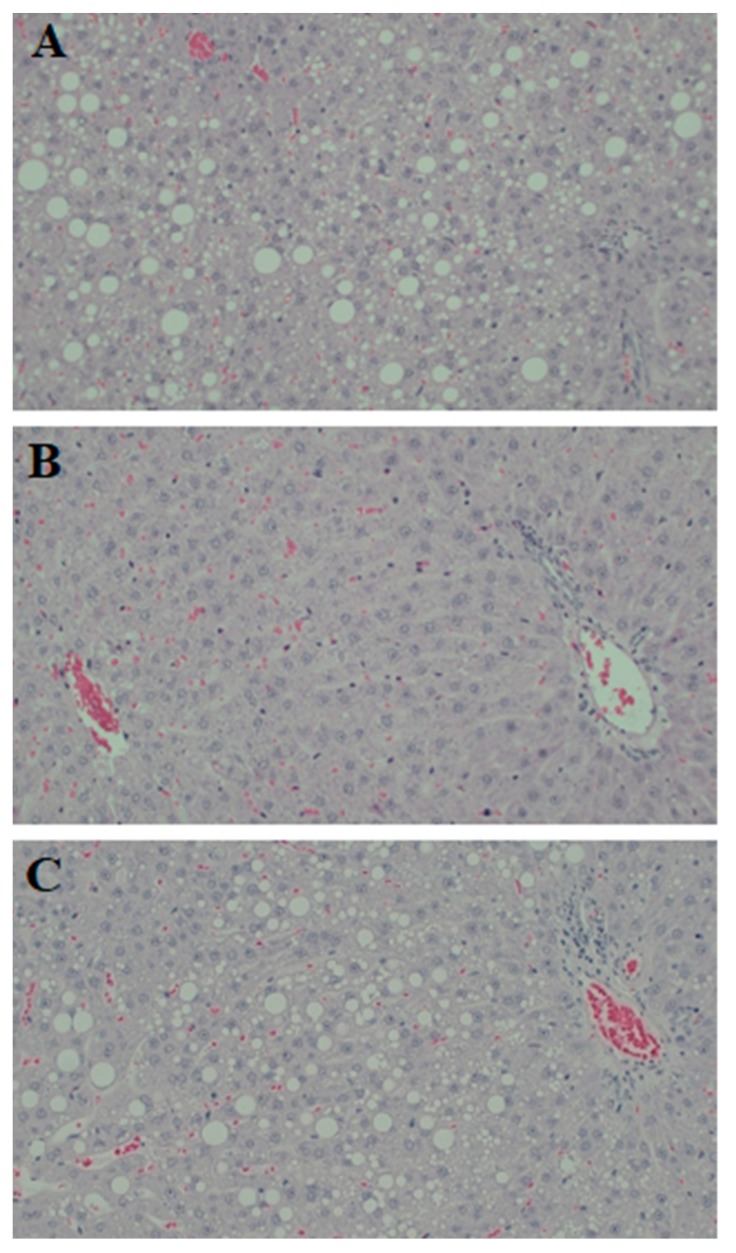
Representative hematoxylin and eosin-stained histological sections of rat liver: (**A**) Samples from the control group showing marked fatty change. There is mixed small- and large-droplet macrovesicular steatosis affecting most hepatocytes. There is no ballooning, inflammation or fibrosis. (**B**) Rat liver sample from the coffee group showing normal morphology with no significant steatosis. (**C**) Marked steatosis, predominantly large-droplet macrovesicular, in most hepatocytes without ballooning, inflammation, or fibrosis in a random sample from the nutraceuticals group.

The blood level of ALT was measured as a marker of fatty liver induced liver cell inflammation and damage. While circulating ALT levels were not affected by either of the two interventions, the liver triglycerides content was lower in the coffee group compared to the control rats (*p* = 0.003) (Table 2). In the microscopic examination of stained liver sections, panacinar steatosis was visible throughout the liver parenchyma in control and nutraceuticals groups, while the cells’ morphology was relatively normal with mild steatosis in samples from the coffee group (Figure 3). Steatosis was dominantly of the macrovesicular type with both small and large droplets. On a scale of 0–3, the mean score of steatosis was 2.13 (SD = 0.83) in the control, 1.38 (SD = 0.74) in the coffee, and 2.00 (SD = 0.89) in the nutraceuticals groups, which shows some degrees of variation. Similarly in all groups, infiltration of inflammatory cells, ballooning, and fibrosis were at a minimal level. No effects of coffee or coffee chemicals were detected on the total plasma antioxidant capacity (Table 2).

### 3.5. In Vivo Hyperpolarized-[1-^13^C]-Pyruvate Metabolic Examinations

**Figure 4:**
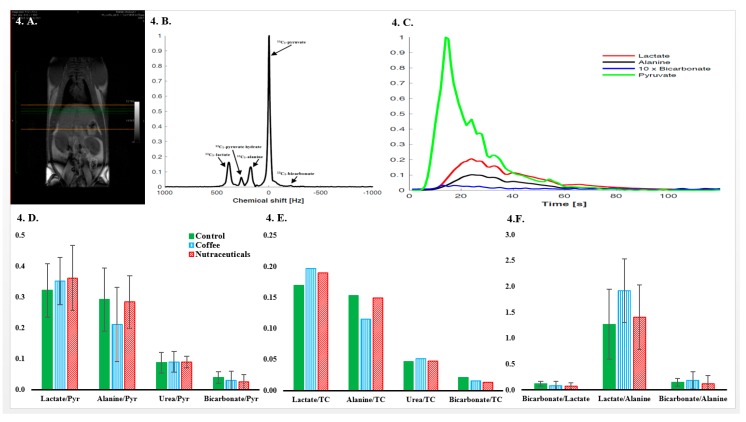
(**A**) A typical full-torso coronal ^1^H MR image; (**B**) a representative 2D ^13^C sum spectra observed after injecting the hyperpolarized [1-^13^C]-pyruvate; (**C**) changes in the signal intensity of metabolites over the 60-s scanning period; (**D**) area under the curve of signal intensity‒time curves of each metabolite normalized to [1-^13^C]-pyruvate and (**E**) total carbon. (**F**) Signal intensity ratios between pyruvate metabolites; error bars represent the standard deviation of the mean.

Four rats randomly selected from each study group were assigned to the MR spectroscopy analysis at intervention week 12. Figure 4A illustrates a typical anatomical image used for positioning the ^13^C imaging plane. After rapid intravenous injection of hyperpolarized-[1-^13^C]-pyruvate to fasted animals, signals from hyperpolarized-[1-^13^C]-pyruvate hydrate, -[1-^13^C]-lactate, -[1-^13^C]-alanine, [1-^13^C]-bicarbonate, and -[^13^C]-urea were detected in the spectra as shown in a representative example in part B of Figure 4. Signal intensity data were generated for each metabolite by computing AUC of signal intensity curves obtained from a 1-min scanning period (see example in Figure 4C). AUC values are presented as proportional to pyruvate and total carbon. The tendency towards lower mean alanine/pyruvate and alanine/total carbon was not statistically significant. The concentration of other metabolites was similar in all groups, as displayed in Figure 4.

### 3.6. Urine 1H-MR Spectroscopy. 

According to Table 2, despite a trend of higher levels in the coffee group, trigonelline excretion in urine was not significantly different between the intervention groups. The measurements showed closely similar levels of ethanol in the urine samples of all study groups (Table 2).

## 4. Discussion

In the current study, a high-fat-/high-sugar-fed model of MetS was employed. This model is among the best animal models of MetS since this Western-type diet can induce several signs and symptoms of human MetS in rodents [29]. Adding fructose to a high-fat diet is shown to intensify its deleterious effects on hepatic steatosis and lipid metabolism [30]. Fructose was added to drinking water, rather than the food, to cover the pungency of coffee brew and balance fluid consumption between groups.

Contrary to the nutraceuticals, whole coffee brew consumption diminished the overall food intake and rate and amount of weight gain caused by HFHF food. Cowan et al. mirrored these results with a higher dosage of instant coffee using a similar animal model [31]. Modest weight modifying effects of coffee have also been observed in some [32,33] but not all [34,35] of the previous comparable studies. Caffeine is known to induce weight loss in rodents [36,37] and help to maintain weight loss in humans [38], but it may not be the only player. CGA-rich green coffee extracts [21,39] and pure CGAs [40,41,42,43] have been proved effective in reducing body weight. Insignificant effects of a phenolic acid-rich chemical mixture may be related to the lower dosage compared to other studies [40,42,43] or different route/frequency of administration [41]. It is likely that in moderate dosages the synergy between caffeine and CGAs plays an important role in reducing weight gain. A comprehensive review of the anti-obesity effects of coffee and their proposed mechanisms has recently been published [44].

In the present study in rats, fasting insulin showed an unsteady increase over time, with an upsurge between weeks 10 and 14 indicating a delayed development of IR, which was alleviated, but not halted, by coffee. This observation, confirmed by a significant reduction in HOMA-IR index, points to insulin-sensitizing effects of coffee and further corroborates the OGTT results. Although the literature is not consistent [31], the present findings correspond to some earlier reports [35,45]. It is puzzling though why the nutraceuticals did not show significant insulin-sensitizing efficacy. The reason might lie in the lower bioavailability of pure chemicals or the lack of other coffee compounds such as lignans and cafestol, which were shown to possess insulinotropic and insulin-sensitizing activities [46,47,48]. Moreover, the role of caffeine should not be neglected. Acute caffeine administration decreases insulin sensitivity [49]; however, in a time span of several weeks, this methylxanthine exhibits enhancing effects on glucose disposal rate [34,37]. Comparable FPG levels in all groups were not surprising based on the knowledge that this index of liver glucose production was either unaltered [32,33] or increased [31] in the former studies. Nevertheless, lower HbA1C in the coffee group signifies a better long-term glycemic control, which is a better predictor of cardiovascular disease and all-cause mortality than the FPG [50].

Noticeably, unfiltered coffee exhibited a significant triglyceride-lowering effect in this study. On the contrary, neither coffee nor nutraceuticals altered the plasma levels of cholesterol and NEFAs after 14 weeks of intervention. Current knowledge suggests that coffee contains compounds with opposing effects on lipoprotein metabolism i.e., diterpenes (cafestol and kahweol) in unfiltered coffee can increase total cholesterol, Low-density lipoprotein cholesterol (LDL-C), and triglyceride levels [51], while CGAs and melanoidins show the opposite effects on both triglycerides and LDL-C [52,53]. In rodents, however, this balance tends to shift towards the lipid-lowering effect since they are relatively resistant to the effects of cafestol [54]. This characteristic may reflect differences in lipoprotein metabolism between rats and humans [55]. An unchanged lipid profile in the nutraceuticals group can be due to the absence of other active substances such as the melanoidins.

Direct measurements showed that coffee administration had reduced the hepatic deposition of triglycerides to almost half compare to the control rats. These results were further corroborated by semi-quantitative histological evaluations. Although this model did not display the advanced features of NAFLD or non-alcoholic steatohepatitis, a significant reduction of ectopic lipid deposition provides evidence for the protective properties of coffee against the development and progression of diet-induced NAFLD. Improvement in different aspects of the HF-diet-induced fatty liver was also documented previously in rats [35,56]. Experimental evidence from human subjects is lacking.

The novel hyperpolarized-^13^C MR spectroscopy technique enabled us to follow the formation of main pyruvate metabolites in living animals. Since pyruvate is at the intersection of energy metabolism, proportional quantification of its metabolites would provide valuable information about the flux of key enzymes namely pyruvate dehydrogenase complex, alanine aminotransferase, and lactate dehydrogenase [57]. To the best of our knowledge, this technique has never been employed in investigating the effects of coffee or coffee chemicals in living animals. This experiment was aimed to explore a possible shift in hepatic carbohydrate metabolism caused by long-term consumption of coffee or its selected compounds. In theory, answering the question of which metabolite conversion rates are likely to be modified by coffee is challenging. However, reversing some of the changes induced by a HF diet appears to be a reasonable response. It was shown that six weeks of HF diet can increase alanine/pyruvate and lactate/pyruvate ratios, accompanied by a surge in serum ALT levels [58]. Therefore, the tendency towards a lower alanine/pyruvate ratio in the coffee group compared to the control can be considered a metabolically favorable effect of coffee in this model. Non-significant differences between groups were probably due to the small sample size of four rats per group. In a previous study, four weeks of metformin therapy increased hepatic lactate/pyruvate but did not alter bicarbonate/pyruvate ratios in male Wistar rats [59]. Future studies on the effects of coffee on intracellular carbohydrate metabolism and their relevance to its metabolic effects are needed.

The following limitations must be taken into consideration when evaluating the presented results. While it needs to be further confirmed, there are a few clues in the literature that pure CGAs may have lower absorption and different excretion behavior from CGAs from coffee [60]. Lack of chemical analysis of the coffee brew used in the present study for chlorogenic acid, trigonnelline, and caffeic acid poses an important limitation in comparing the two intervention groups, even though the dose estimation had been performed based on high-quality published papers. It is, however, not likely that the insignificant results obtained in the nutraceuticals group can mainly be caused by differences in the concentration of compounds between the intervention groups. The absence of standardized degrees of roasting, which affects the CGAs and trigonelline content of coffee, in commercialized products reduces the reproducibility of coffee studies. Next point is that administering coffee and nutraceuticals in water makes it difficult to deliver the exact dosage and increases the probability of degradation and sedimentation (especially in case of caffeic acid). Furthermore, the poor solubility of caffeic acid in water necessitated the use of ethanol as a solvent. Accordingly, each rat received ~0.6 g of ethanol per day equal to 1.5 European standard drinks in a human. As described before, urine ethanol levels evidenced equal consumption in all groups, which eliminated the possibility of affecting our results. Ironically, including moderate amounts of ethanol in the rats’ regime might have made it more similar to a typical Western diet. Finally, the combination used in the current study was one of the many conceivable combinations of coffee compounds and not necessarily the most bioactive one. Multiple combinations need to be studied simultaneously in order to find the compounds with synergistic effects.

## 5. Conclusions

The current study using a model of diet-induced MetS showed that a moderate dosage of unfiltered coffee can reduce food intake and weight gain in addition to mitigating the average glycemia and IR compared to water. Coffee also diminished circulating triglycerides and their deposition in the liver (liver steatosis). Consuming a comparable dosage of a combination of coffee nutraceuticals (CGA, caffeic acid, and trigonelline) did not replicate any of the metabolic effects of coffee. In summary, these findings confirmed the previous research on the efficacy of coffee in improving various components of the MetS, which may be important contributors to the overall positive health effects of coffee. Our observations also suggest that hydroxycinnamic acids and trigonelline are not the only crucial compounds involved in the biological effects of coffee. For future research, it is proposed to investigate more complex combinations of coffee compounds including caffeine and/or cafestol at higher dosages.

## Figures and Tables

**Table 1:** Mean (SD) of glucose and insulin measures recorded four times during the intervention period; AUC: area under the curve of repeated measurements. Significant one-way ANOVA *p*-values are in bold.

Mean (SD)	Control	Coffee	Nutraceuticals	Between Group *p*-Value
Fasting plasma glucose (mmol/L)				
Baseline	7.69 (0.75)	7.35 (0.42)	7.60 (0.82)	0.588
Week 4	8.24 (1.09)	7.93 (1.03)	8.43 (0.63)	0.560
Week 10	8.17 (1.92)	7.51 (0.61)	7.70 (0.92)	0.588
Week 14	10.00 (1.48)	9.09 (1.50)	9.46 (1.55)	0.491
Glycated hemoglobin (mM/M)				
Week 14	22.88 (1.25)	21.25 (1.04) *	23.67 (2.16) ^††^	**0.018**
Fasting plasma Insulin (mU/L)				
Baseline	24.47 (8.79)	23.50 (7.88)	26.94 (5.58)	0.647
Week 4	52.55 (21.29)	34.26 (13.49)	52.85 (23.56)	0.126
Week 10	41.21 (13.82)	26.49 (6.14)	43.31 (8.88) ^††^	**0.008**
Week 14	80.73 (33.83)	53.11 (29.25)	76.07 (27.91)	0.211
AUC	146.38 (49.54)	100.79 (34.09) *	133.16 (41.80) ^†^	**0.049**

* significantly different from the control group (* *p* ˂ 0.05); † significantly different from the other intervention group († *p* ˂ 0.05, †† *p* ˂ 0.01); Statistically significant *p*-values are shown in bold.

**Table 2:** Mean (SD) value of endpoint immunoassay and ^1^H-MR spectroscopy measurements. F-test *p*-values are presented in the right-most column. Significantly different values from the control and from the other intervention group are bolded and marked by superscript symbols.

Mean (SD)	Control Group	Coffee Group	Chemicals Group	Between Group *p*-Value
Plasma				
Lipids (mmol/L)				
Total Cholesterol	1.650 (0.118)	1.728 (0.099)	1.558 (0.125)	0.604
NonHDL-C	0.544 (0.045)	0.535 (0.092)	0.488 (0.029)	0.588
LDL-C	0.250 (0.023)	0.336 (0.036)	0.257 (0.011)	0.156
HDL-C	1.106 (0.085)	1.193 (0.098)	1.080 (0.104)	0.690
Triglycerides	0.649 (0.041)	0.380 (0.053) **	0.535 (0.082)	**0.010**
Nonesterified fatty acids	0.279 (0.039)	0.295 (0.025)	0.288 (0.039)	0.941
Adiponectine (μg/mL)	5.694 (0.371)	5.188 (0.618)	4.701 (0.573)	0.474
Total antioxidant capacity (Trolox equivalent, mmol/L)	102.323 (1.743)	97.228 (2.168)	102.272 (2.344)	0.152
Alanine transaminase (U/L)	18.838 (1.280)	19.875 (1.612)	17.983 (1.061)	0.718
Liver triglycerides content (mmol/L)	2.806 (0.384)	1.379 (0.108) **^,†^	2.386 (0.432)	**0.010**
Urine (relative units)				
Trigonelline	0.0002 (0.000)	0.038 (0.015)	0.025 (0.019)	0.597 ^¥^
Ethanol	1.355 (0.829)	1.378 (0.257)	1.404 (0.262)	0.991

** significantly different from the control group (** *p* ˂ 0.01); † significantly different from the other intervention group († *p* ˂ 0.05); ¥ independent *t*-test *p*-value of comparison between two intervention groups; Statistically significant *p*-values are shown in bold.

## Appendix A

**Table A1:** Nutrient composition of the high fat diet, D12492 (Research Diets, Inc., New Brunswick, NJ, USA), used in the current study in combination with 20% W/W fructose in drinking water to induce a rat model of metabolic syndrome.

Ingredients	Gram Percent
Casein, 30 Mesh	25.84
L-Cystine	0.39
Corn Starch	0.00
Maltodextrin 10	16.15
Sucrose	8.89
Cellulose	6.46
Soybean Oil	32.53
Lard	31.66
Mineral Mix S10026	1.29
DiCalcium Phosphate	1.68
Calcium Carbonate	0.71
Potassium Citrate, 1 H_2_O	2.13
Vitamin Mix V10001	1.29
Choline Bitartrate	0.26
